# Ozone Sensing Based on Palladium Decorated Carbon Nanotubes

**DOI:** 10.3390/s140406806

**Published:** 2014-04-14

**Authors:** Selene Capula Colindres, Khalifa Aguir, Felipe Cervantes Sodi, Luis Villa Vargas, José A. Moncayo Salazar, Vicente Garibay Febles

**Affiliations:** 1 Instituto Mexicano del Petróleo, Eje Central Lázaro Cárdenas 152, 07730 D.F., Mexico; E-Mail: vgaribay@imp.mx; 2 Aix-Marseille Université, CNRS, UMR 7334, Av. E.N. Niémen 13397, Marseille, France; E-Mail: khalifa.aguir@im2np.fr; 3 Departamento de Física y Matemáticas, Universidad Iberoamericana, Prolongación Paseo de Reforma 880, Lomas de Santa Fe, 011219 D.F., Mexico; E-Mails: felipe.cervantes@ibero.mx (F.C.S.); armandomoncayo@gmail.com (J.M.S.); 4 Instituto Politécnico Nacional, Centro de Investigación en Computación, Av. Juan de Dios Batiz s/n Col. Nueva Industrial Vallejo 07738 D.F., Mexico; E-Mail: lvilla@cic.ipn.mx

**Keywords:** gas sensor, ozone, multiwall carbon nanotubes, Pd nanoparticles

## Abstract

Multiwall carbon nanotubes (MWCNTs) were easily and efficiently decorated with Pd nanoparticles through a vapor-phase impregnation-decomposition method starting from palladium acetylacetonates. The sensor device consisted on a film of sensitive material (MWCNTs-Pd) deposited by drop coating on platinum interdigitated electrodes on a SiO_2_ substrate. The sensor exhibited a resistance change to ozone (O_3_) with a response time of 60 s at different temperatures and the capability of detecting concentrations up to 20 ppb. The sensor shows the best response when exposed to O_3_ at 120 °C. The device shows a very reproducible sensor performance, with high repeatability, full recovery and efficient response.

## 1. Introduction

Currently, gas sensors with optimized features such as low cost, fast response, high gas selectivity and sensitivity, good stability and small size are required. Carbon nanotubes (CNTs), nanowires and graphene have been recently used for this purpose with good results [1–5]. In particular, CNTs exhibit unique properties for their application as gas sensors. Beside their high surface to volume ratio, which means a large area for gas interaction [6]; CNTs present an extreme sensitivity to charge transfer and chemical doping effects by the interaction with various molecules [7,8]. Electrical properties of p-type carbon nanotubes are modified when oxidizing or reducing gas molecules that adsorb and interact with them. Adsorbant molecules change the density of main charge carriers in nanotubes, altering their conductance [8,9]. Finally, CNT-based gas sensors are able to work at room temperature with low power consumption. This characteristic is useful in hazardous applications when continuous monitoring of gases is necessary [10].

In order to increase the reactivity of CNTs, metal nanoparticles (NPs) are frequently incorporated along their structures, acting as active sites during the interaction between target analytes and CNTs' walls [10–12]. The interest of using metal NPs is due to their high catalytic activity, adsorption capacity and efficient charge transfer [13]. Metal NPs are highly sensitive to changes in their environment, providing a wide range of reactivity towards different gases. The selectivity and sensitivity of sensors can be tuned using several types of NPs because their reactivity depends on the element constituting the NPs. The key concept in CNTs decorated with nanoparticles (CNTs-NP) is that upon adsorption of a target molecule, a significant amount of charge is donated or accepted by the NPs, and as a result the electron transport in the CNTs is affected and this change is easily measureable [10].

Ozone is a principal cause of photochemical smog and atmospheric contamination. It is harmful to human health; breathing ozone-containing air reduces lung function, aggravating asthma and other respiratory conditions [14]. Furthermore, high ozone levels can also harm sensitive vegetation in forested ecosystems [14]. The standard method for ozone detection is based on a UV adsorption method [15]. Accuracy and high sensitivity are reported for this method, however, it presents drawbacks such as complexity of the apparatus with high cost and large detector size. Due to their compact size, adsorption capacity, wide range of reactivity of several gases, simplicity in fabrication, operation in harsh environmental and at high temperatures sensors base CNT-nanoparticles are an alternative to replace the conventional method for detection of ozone.

Few works have reported the detection of ozone gas molecules with CNTs used as the sensitive material [15–18]. Park *et al.* [15] showed the response of a sensor using single wall CNT (SWCNT) networks; their sensor was sensitive to ozone down to 50 ppb with a rapid response as well as a fast recovery. Wongwiriyapan *et al.* [16] reported ozone sensitivity when using SWCNT networks directly grown on a conventional sensor substrate. Ozone was detected down to 6 ppb at room temperature while operating with a fast response. Ghaddab *et al.* [17] compared the gas sensing properties between three types of materials: SWCNT, SnO_2_ and SWCNT/SnO_2_ hybrid materials. Among these, the latter were significantly more responsive to ozone and ammonia than pure SnO_2_ or SWCNTs. The detection limit at room temperature was evaluated to be lower than 20 ppb.

To the best of our knowledge, although Pd decorated CNTs have been used for hydrogen detection [19,20], it has not been used for ozone sensing before. A key step for the production of CNTs-Pd is the NP attachment to the CNTs. Different strategies have been employed to promote the incorporation of metal nanoparticles on CNT walls such as precipitation from a metal salt solution [21], chemical attachment of preformed clusters [22], electron beam evaporation [2,10], sputtering coating [23,24], chemical functionalization [18], thermal evaporation [20], electrochemical functionalization [19] drop-coating [25] and vapor phase impregnation decomposition process (VPID) [26]. The last one has been tested as a good method for incorporation of metallic particles. It showed successful incorporation of nanoparticles on the surface of titania nanoparticles and CNTs [26] with homogeneously decoration and narrow particle size distribution.

In this work, MWCNT were decorated by VPIDM with Pd NPs and the MWCNT-Pd sensitive material was used to detect ozone at concentrations ranging from 20 ppb to 300 ppb, in a temperature interval from room temperature to 200 °C.

## 2. Experimental Section

### 2.1. Synthesis and Purification of MWCNTs

MWCNT were produced by chemical vapor deposition (CVD) as previously reported [27] whereby microdroplets of a ferrocene/toluene solution at 3.5/9.6 wt% were supplied by an ultrasonic atomizer device (pyrosol 7901, RBI, Meylan, France) connected to a quartz tube reactor. Ar (99.99% purity) was used as the carrier gas with a flow rate of 2.5 L/min. After 40 min. of CVD reactor at 850 °C the system was allowed to cool down to room temperature for about 120 min.

Morphological characteristics of the as-obtained nanotubes were determined by high resolution transmission electron microscopy (HRTEM) in a Tecnai G2 F30 instrument (FEI Company, Hillsboro, OR, US); Raman spectra were obtained by a LabRAM HR800 Raman spectometer (Horiba Jobin Yvon, Villeneuve d'Ascq, France) with an excitation wavelength of 633 nm.

Generally, the as-prepared CNTs contain impurities like metal catalysts and amorphous carbon, which could make it difficult to understand and monitor the intrinsic properties of the nanotubes [28,29], and could affect the behavior of any device that is based on them [30]. The purification process is a fundamental step to eliminate such impurities. In this study, the sample of CNTs was stirred in a 1:3 solution of sulphuric and nitric acid for 5 h at 70 °C. After this treatment, the resulting product was washed with distilled water several times, followed by filtration. Finally, the sample was dried at 60 °C for 6 h [26].

### 2.2. Pd Decoration of MWCNTs

As mentioned before, palladium NPs were incorporated on the surfaces of the CNT's by the VPID method [26]. For the preparation, CNTs and Pd(acac)_2_ were mechanically mixed for 15 min until the mixture was homogenous. Then they were kept at a constant temperature of 180 °C for 10 min under 66.6 kPa pressure, inside a horizontal quartz-tube reactor with argon gas (3 × 10^−6^ m^3^/s). Next, the product was moved to a raised temperature zone (400 °C) in order to induce the precursor decomposition. By this procedure CNTs were functionalized or decorated with 3 wt% of Pd NPs.

### 2.3. Sensor Assembly

**Figure 1.**
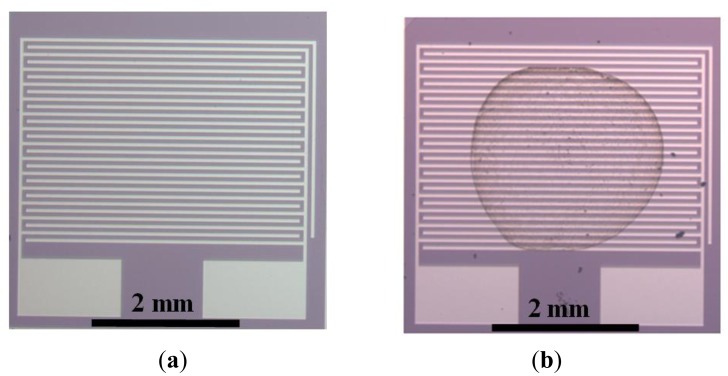
Images of the device: (**a**) two interdigitated platinum electrodes on silicon dioxide; (**b**) device with Pd deposited by drop coating.

The sensor consists of two interdigitated platinum electrodes, obtained by standard pulverization method on silicon dioxide in a resistor configuration (Figure 1a). MWCNTs-Pd was dispersed in glycerol by ultrasonication for 1 h at room temperature [10]. The drop coating method was used to deposit the glycerol solution on the device. The deposited drop was dried at 200 °C for 2 h to eliminate the glycerol (Figure 1b).

### 2.4. Gas Response Measurements

The gas sensing properties of MWCNTs-Pd film were investigated in a sealed stainless test cell in order to control the different temperatures. The sensor was exposed to air with different concentrations of ozone (20, 50, 100, 200 and 300 ppb) during 60 s and finally exposed to a clean air flow for recovery. The gas mixtures were generated by an InDevR (2B Technologies, Boulder, CO, USA) calibrated commercial ozone generator. This generator is designed to provide accurate, precise and constant amounts of O_3_ produced by UV irradiation of an air flow and electrical measurements (electrical resistance *versus* time) were monitored by a KEITHLEY 6430 digital multimeter (Keithley, Panama City, FL, USA) connected to the personal computer.

## 3. Results and Discussion

### 3.1. Transmission Electron Microscopy (TEM) Characterization

**Figure 2.**
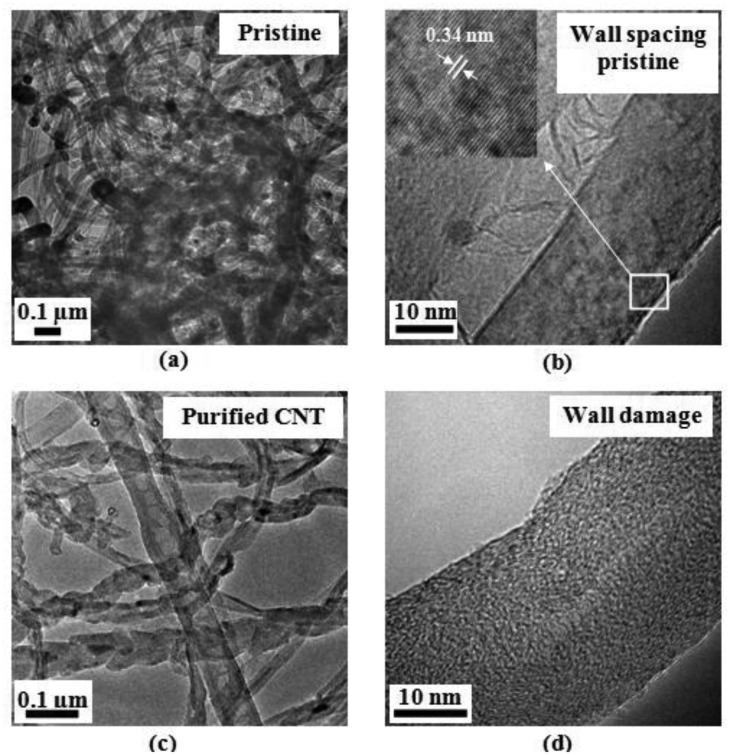
TEM images of MWCNT at different magnifications; (**a**) as-prepared nanotubes (pristine); (**b**) HRTEM of the graphitic layer of the CNT walls; (**c**) acid treated CNTs and (**d**) damage produced by purification at the walls of CNTs.

Figure 2a,b presents TEM images of pristine as prepared MWCNTs at low and high magnification. The MWCNTs reveal “nanofiber” features. The average diameter of the MWCNTs was found to be 52.7 nm and they were several hundred microns long. Figure 2b presents the HRTEM of tubes walls at low magnification, revealing their multiwalled nature. The inset of Figure 2b shows the graphitic layers with wall spacings of 0.34 nm between them.

Figure 2c,d shows the TEM micrographs of acid treated MCWNTs. After purification most of the impurities are removed, and MWCNTs present damage on their walls with alteration of the external morphology. Figure 2c shows wall distorted MWCNTs attributed to a higher concentration of defects which leaves functionalized holes with oxygenated functional groups such as carboxylic acid, ketone, alcohol, and ester groups; all of them facilitating the oxidation [31]. Oxidants breach the carbon shell and then oxidize the metal catalysts to the corresponding metal oxide or hydroxide, when the metal is oxidized, the volume increases and the metal oxides crack open the carbon coating [28]. Figure 2b presents a sample of as-fabricated nanotubes and is clearly observed that the number of walls is different in comparison with the sample after purification (Figure 2d); the walls were altered until they achieve an amorphous morphology.

**Figure 3.**
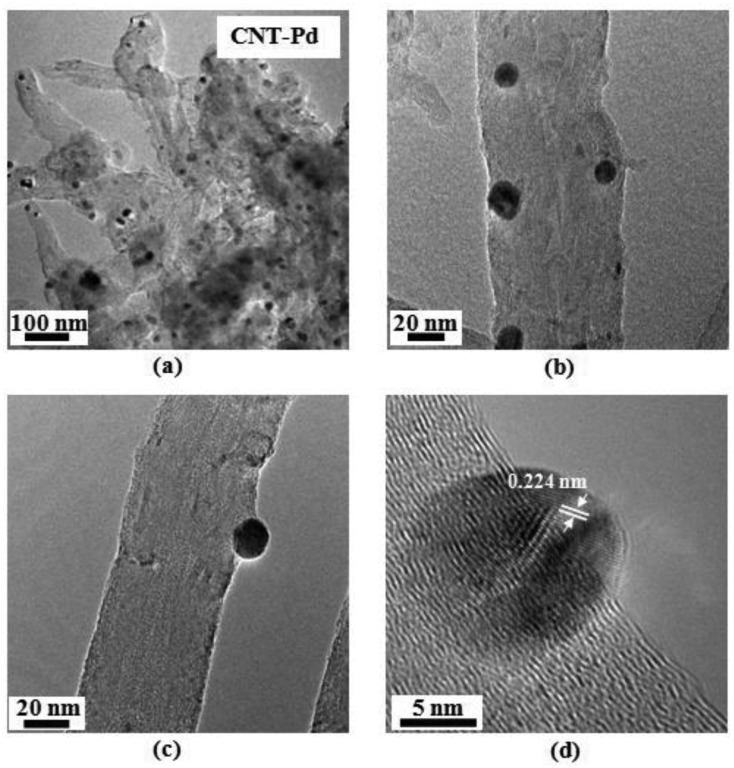
TEM images. (**a**) MWCNT-Pd; (**b,c**) individual MWCNT decorated with Pd nanoparticles; and (**d**) HRTEM image of a Pd nanoparticles. The lattice spacing is measured as 0.224 nm, corresponding to the (111) plane of Pd with FCC crystal structure.

TEM images of Pd decorated CNTs are shown in Figure 3. Heterogeneous nanotubular morphology with the tubular structure is partially destroyed by the aggressive acid treatment is shown in Figure 3a. Pd NPs were found on the outside walls and the tips of MWCNTs. No agglomeration is observed after the decoration process. Defects serve as anchor by attachment of functional groups and NPs. The HRTEM image in Figure 3b shows individual MWCNTs with incorporation of NPs with average size of about 10.8 nm. Figure 3d shows a HRTEM image of a Pd nanoparticle on the damaged nanotube wall. The lattice interspacing measurement was 0.224 nm, corresponding to FCC crystal of Pd (JCPD-0681).

### 3.2. Raman Characterization

**Figure 4.**
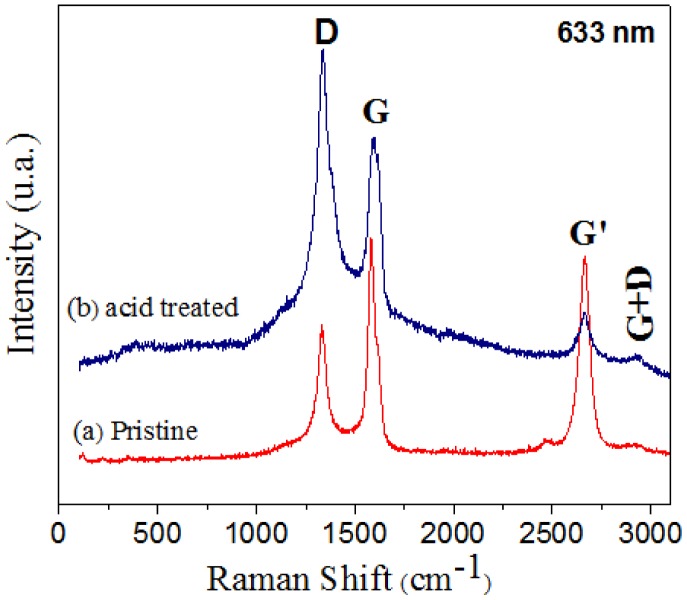
Raman spectrum of CNTs using a laser excitation of 633 nm, (**a**) pristine sample; and (**b**) acid treated.

Figure 4 shows the Raman spectra of pristine and acid treated MWCNTs. Both sample spectra present four characteristic bands: mode D (1,333 cm^−1^), mode G (1,576 cm^−1^), mode G' (2,662 cm^−1^), and mode G+D (2,919 cm^−1^) [32–34], respectively. By comparing the spectra in Figure 4a,b it seems that purification leads to an increase in defects.

The increasing intensity of the D band in the acid treated MWCNTs (Figure 4b) is due to the presence of a high density of defects (vacancies and heptagon-pentagon pairs) on the tube walls [32–34]. The D and G mode intensities have usually been employed as an indication of chemical modification of CNT [35] or degree of “graphitization”. The relative intensities between G and D modes, (I_G_/I_D_), are I_G_/I_D_ = 1.57 and I_G_/I_D_ = 0.79. The intensity ratio of the D to G-band decreases in the case of the purified samples. This could be attributed to the destruction of graphitic integrity and the subsequent formation of small graphitic fragments [36].

### 3.3. CNT-Pd Sensor Response

The detection response of our devices is measured using the electric resistance response, normalized by S(%) = R_g_ − R_0_/R_0_ × 100, where R_g_ represents the ozone+air mixture resistance interaction with the sensitive material, and R_0_ stands for the air resistance [18,19].

**Figure 5.**
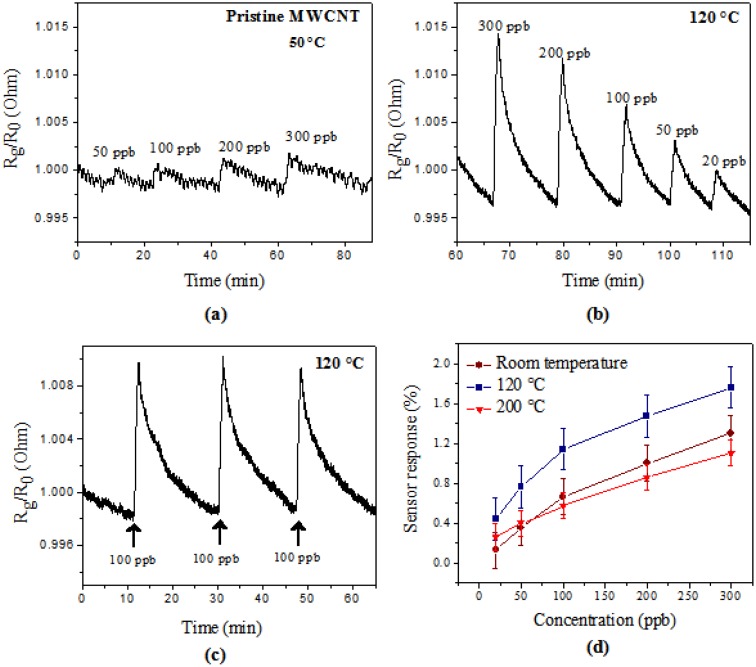
Electrical resistance change of sensors in the presence of O_3_, (**a**) pristine MWCNT tested at 50 °C with different concentrations; (**b**) MWCNT-Pd evaluation at different concentrations in the range 20 to 300 ppb; (**c**) repeatability of response sensor MWCNT-Pd at 100 ppb; and (**d**) comparison of the calibration curves at different MWCNT-Pd sensor temperatures.

Figure 5a shows the response to O_3_ for the pristine MWCNT-based sensor. Comparing the sensor responses of the different materials, pristine MWCNTs present lower responsiveness (0.44% to 300 ppb and 0.34% to 50 ppb at 120 °C). This behavior is due to the presence of impurities having negative effects on the inherent properties of CNTs [29,37,38]. It has been reported that impurities can change the electronic and electrochemical behavior of nanotubes [39]. A study of a pristine CNT-based sensor reports slow and incomplete recovery [1,40]. Kong *et al.* [1] developed a field effect transistor (FET) with SWCNTs with an extended recovery time of 12 h, at room temperature after gas exposure to NO_2_ and NH_3_. Similarly, SWCNT-based sensors used to detect organic vapors at room temperature were manufactured by Li *et al.* [40]; the recovery time was on the order of 10 h. Additionally, the hydrophobicity and chemical inertness of pristine CNT causes low selectivity and in some cases null sensitivity to many types of molecules. This behavior is due to a strong sp^2^ C-C bonding, in comparison with the weak interaction between CNT walls and gas molecules [2,8,41–44]. All these disadvantages limit the applicability for sensing purpose of pristine CNTs.

The measurements for the detection behavior of sensors made of purified detection MWCNT (without NPs) and MWCNTs-Pd are compared in Table 1. The purified MWCNT-based sensor shows a higher responsiveness at both tested temperatures. However, the sensor response dramatically decreases when the sensor is exposed to a second cycle of O_3_. This behavior is probably due to saturation of adsorption sites by the occupation of all initially free adsorption sites [45]. In the case of the MWCNT-Pd based sensor, the response was independent of the number of exposure cycles or the time lapse between the sensor fabrication and its use. Due to this, we continued our experiments using only the MWCNTs-Pd-based sensor, which sensor resistance response is shown in Figure 5.

The MWCNT-Pd based sensor electrical response evaluated at 120 °C at different concentrations is summarized in Figure 5b. The sample showed sensitivity to ozone from a low concentration (20 ppb), and the resistance showed an increase when the concentration of gas increases. These results provide evidence that the MWCNTs-Pd sensor has satisfactory sensitivity to O_3_ detection under the test conditions. The reproducibility response of the sensor at 100 ppb is observed in Figure 5c. The resistance increases when the sensor is exposed to ozone gas (injection started), and the resistance decreases in contact with the air flow (injection stopped). The sample presents a response time of less than 1 min and a recovery time of about 20 min. Figure 5d shows the calibration curves of the MWCNTs-Pd film exposed to ozone at different temperatures. The best response was found at 120 °C.

This sensor presents a decrease of the resistance in the presence of an oxidizing gas, indicating a p-like behavior, a result that is in good agreement with previous studies [15,17]. Park *et al.* [15] and Ghaddab *et al.* [17] using SWCNT and hybrid tin dioxide/CNT as O_3_ sensors reported the mechanisms for the change in the behavior resistance when interacting with an oxidizing gas such as ozone. They mentioned that during gas adsorption, there is an electron charge transfer whereby O_3_ accepts electrons from CNT and the concentration of conducting holes increases experimentally leading a decrement of the signal resistance.

Our MWCNTs-Pd sensor response can be attributed to the following factors: (1) the high surface to volume ratio of the sensitive material (MWCNTs). Wang *et al.* [37] identified four sites of adsorption in SWCNTs: the surface (external surface of the nanotube), channels (interstitial holes formed between the outside of adjacent tubes), pores (inside the nanotubes) and grooves (holes formed between threes of adjacent tubes); (2) Chemical modification caused by the purification processes. Chemical modification can improve solubility, processability, enhance the reactivity and selectivity of CNT surfaces and the selectivity to specific gases [2,10,24,46]. This behavior is attributed to the presence of defect sites generated by the damage to the nanotube framework, which leaves holes functionalized with oxygenated functional groups [31,43]. The functional groups can act as anchor in the formation and deposition of NPs [12]; (3) Charge transfer between gas molecules and CNT and Schottky barrier modulation. Battie *et al.* [37,47] have demonstrated that the sensing mechanism depends on the density and film thickness of SWCNT *i.e.*, a high density of nanotubes can promote a decrease in the influence of the Schottky barrier between the metal electrode and the SWCNTs contact. Moreover, when the inter-electrodes distance is lower than 10 μm, there is a probability to obtain metallic nanotubes chains between the two electrodes, that which is why our sensor has an inter-electrode spacing of 50 μm, which reduces the probability of finding CNTs bridging the electrodes. The same author claimed that below the percolation threshold of metallic nanotubes, the transport properties are dominated by Schottky barrier modulation at the interface between the electrodes and semiconducting-nanotubes [47] and (4) the agglomeration of the Pd NPs. The behavior of our MWCNTs-Pd sensor is different from that reported by several previous studies [10,18,24]. These studies mention that the incorporation of NPs increases the selectivity and sensitivity. In our case, MWCNTs-Pd showed moderate sensitivity to ozone. This behavior is probably due to the agglomeration of the metal on the CNT surfaces. In our study, the size of Pd NPs deposited on MWCNTs was about 11 nm in contrast with other studies using the same method of decoration with NPs for CNTs and titania nanotubes, which reported NP diameters about 3.2 and 2.1 nm, respectively [26]. Liang *et al.* [48] deposited Pd NPs on carbon nanofibers (CNFs), obtaining NPs with diameters ranging from 2 to 4 nm. Nanoparticles of noble metals such as Pt, Au and Pd are highly mobile and they migrate to the surface and agglomerate into larger particles due to weak interactions between metal and support [49]. Although it is known that functional groups help to avoid agglomeration of NPs, in our case, agglomeration was possible because the functional groups form bonds with CNTs through weak interaction. The metal particle size plays an important role: generally, small Pd particles with a uniform size distribution are favorable for the activity and selectivity of different reactions [48].

In previous works on O_3_ sensing with CNTs, mainly SWCNTs have been used [15–17]. The response of a SWCNT sensor to 50 ppb was 11.1% and reached a maximum of 14.1% to 1 ppm with a response time of 100 s. Both results of the SWCNT sensor were reported after thermal treatment [15–17]. The sensor was saturated nearly 200 s after being exposed to the first concentration. The responsiveness of SWCNT sensor directly grown on a conventional substrate, detected down to 6 ppb at room temperature [16]. The detection efficiency of a hybrid SnO_2_/SWCNT sensor was reported to be ozone detection at ≤21.5 ppb (∼3% sensor response) and its maximum at 290 ppb (∼13% sensor response) at room temperature with a response time of 2,400 s [17]. In [50], plasma enhanced CVD grown MWCNTs showed a resistance change to the presence of O_3_ at concentrations ranging from injection of 25 to 175 ppb, but the sensor response was not sufficiently fast to recover the base line (12,000 s).

## 4. Conclusions

The vapor impregnation-decomposition method was successfully used for incorporation of Pd nanoparticles on the walls of acid treated CNTs. The acid method for CNTs functionalization is efficient, but this process causes damage in the walls of the nanotubes. The MWCNT-Pd sensor was sensitive to O_3_ with high repeatability and sensitive to different concentrations. The best response was found when operated at 120 °C. Our ozone sensor showed an efficient response 60 s after exposure to O_3_, while other works report between 100 to 1,200 s are needed to detect ozone.

## Figures and Tables

**Table 1.** Responsiveness of the purified MWCNT and MWCNT-Pd.

**Sensor Response (%) to O_3_**								

**Material**	**Sensor Operated at 120 °C**				**Sensor Operated at 200 °C**			

	**300 ppb**	**200 ppb**	**100 ppb**	**50 ppb**	**300 ppb**	**200 ppb**	**100 ppb**	**50 ppb**
Purified MWCNTs	4.06	3.43	2.32	1.13	5.0	4.20	3.34	2.19
MWCNTs-Pd	0.76	1.14	1.47	1.76	1.1	0.86	0.57	0.4
